# Bcl-2 regulates store-operated Ca^2+^ entry to modulate ER stress-induced apoptosis

**DOI:** 10.1038/s41420-018-0039-4

**Published:** 2018-02-26

**Authors:** Wen-Tai Chiu, Heng-Ai Chang, Yi-Hsin Lin, Yu-Shan Lin, Hsiao-Tzu Chang, Hsi-Hui Lin, Soon-Cen Huang, Ming-Jer Tang, Meng-Ru Shen

**Affiliations:** 1 Department of Biomedical Engineering, National Cheng Kung University, Tainan, 701 Taiwan; 2 Institute of Basic Medical Sciences, National Cheng Kung University, Tainan, 701 Taiwan; 3 Department of Pharmacology, National Cheng Kung University, Tainan, 701 Taiwan; 4 Department of Physiology, National Cheng Kung University, Tainan, 701 Taiwan; 5 Department of Obstetrics and Gynecology, Chi Mei Medical Center, Liouying Campus, Tainan, 736 Taiwan

## Abstract

Ca^2+^ plays a significant role in linking the induction of apoptosis. The key anti-apoptotic protein, Bcl-2, has been reported to regulate the movement of Ca^2+^ across the ER membrane, but the exact effect of Bcl-2 on Ca^2+^ levels remains controversial. Store-operated Ca^2+^ entry (SOCE), a major mode of Ca^2+^ uptake in non-excitable cells, is activated by depletion of Ca^2+^ in the ER. Depletion of Ca^2+^ in the ER causes translocation of the SOC channel activator, STIM1, to the plasma membrane. Thereafter, STIM1 binds to Orai1 or/and TRPC1 channels, forcing them to open and thereby allow Ca^2+^ entry. In addition, several anti-cancer drugs have been reported to induce apoptosis of cancer cells via the SOCE pathway. However, the detailed mechanism underlying the regulation of SOCE by Bcl-2 is not well understood. In this study, a three-amino acid mutation within the Bcl-2 BH1 domain was generated to verify the role of Bcl-2 in Ca^2+^ handling during ER stress. The subcellular localization of the Bcl-2 mutant (mt) is similar to that in the wild-type Bcl-2 (WT) in the ER and mitochondria. We found that mt enhanced thapsigargin and tunicamycin-induced apoptosis through ER stress-mediated apoptosis but not through the death receptor- and mitochondria-dependent apoptosis, while WT prevented thapsigargin- and tunicamycin-induced apoptosis. In addition, mt depleted Ca^2+^ in the ER lumen and also increased the expression of SOCE-related molecules. Therefore, a massive Ca^2+^ influx via SOCE contributed to caspase activation and apoptosis. Furthermore, inhibiting SOCE or chelating either extracellular or intracellular Ca^2+^ inhibited mt-mediated apoptosis. In brief, our results explored the critical role of Bcl-2 in Ca^2+^ homeostasis and the modulation of ER stress.

## Introduction

Deregulation of apoptosis can lead to cancer and to autoimmune and degenerative diseases^1^. The first identified apoptotic regulator was Bcl-2. The Bcl-2 family of proteins decide the fate of cells with response to survival and death. The proteins of the Bcl-2 family are characterized by homology domains BH1–4 (for Bcl-2 homology domain 1–4). The family can be subdivided in two major groups: the anti-apoptotic subgroup (for example, Bcl-2 and Bcl-xL) and the pro-apoptotic subgroup comprising Bax-like proteins (for example, Bax and Bak), which contain the BH1–3 domains, and the BH3-only proteins (for example, Bid and Bad)^2^. Bcl-2 plays an important role in mitochondria and endoplasmic reticulum (ER)^3–6^. Most of the Bcl-2 family proteins contain a hydrophobic C-terminal domain, required for their specific localization to different subcellular compartments, such as the ER, mitochondria, and perinuclear membranes^7,8^. In the ER, Bcl-2 interferes with the induction of apoptosis by Bax^9^, ceramides, ionizing radiation^10^, serum withdrawal, and c-myc expression^11^. Recently, the focus of researchers has shifted toward finding the possible association between the effects of the Bcl-2 family on Ca^2+^ homeostasis and their role in the control of apoptosis^12,13^. In addition, the specific localization of Bcl-2 in the ER membrane indicates that Bcl-2 regulates filling of ER intracellular Ca^2+^ store^14,15^, suggesting that Ca^2+^ signaling might be a target of the Bcl-2 oncoprotein.

The anti-apoptotic activity of Bcl-2 is mediated by its regulation of handling Ca^2+^ level in the ER and mitochondria. However, determining whether Bcl-2 increases or decreases the ER luminal Ca^2+^ will reveal its true role in the ER Ca^2+^ handling^16^. One hypothesis states that Bcl-2 decreases the Ca^2+^ concentration within the ER such that less Ca^2+^ is available for release into the cytosol, thereby leading to a more modest mitochondrial Ca^2+^ uptake. Many studies show that Bcl-2 can protect cells from stress-induced Ca^2+^ release from ER and lead to subsequent cell death by lowering the loading of Ca^2+^ in the ER^17,18^. In contrast, it has been argued that Bcl-2 does not diminish the content of Ca^2+^ pool, instead it inhibits the opening of inositol 1,4,5-trisphosphate receptors (IP3Rs) on the ER, thereby reducing the extent of Ca^2+^ mobilization for a given magnitude of cell stimulation^19^. He et al. reported that Bcl-2 mediated Ca^2+^ uptake and preserved the Ca^2+^ pool of the ER to prevent depletion of the pool^14^. Furthermore, Bcl-2 overexpression is associated with the reduction in the transient elevation of cytosolic Ca^2+^ induced by thapsigargin (TG)^20^. A proposal common to many of these studies is the proposal that Bcl-2 functions to reduce the magnitude of increase in cytosolic Ca^2+^ concentration in response to apoptotic stimuli. However, the two different functions of ER-resident Bcl-2 may possibly depend on different cell contexts; this relation needs to be clarified in detail.

Ca^2+^ can serve as an apoptotic signaling factor when delivered at the wrong time and to the wrong place^21,22^. Disruption of intracellular Ca^2+^ homeostasis by influx of extracellular Ca^2+^ is always lethal to cells^23^. It was believed that Ca^2+^-related cell death could be triggered by large, sustained increases in cytosolic Ca^2+^. More specifically, both persistent Ca^2+^ release from the ER and store-operated Ca^2+^ entry (SOCE) through Ca^2+^ release-activated Ca^2+^ channels are apoptogenic^24,25^. Several anti-cancer drugs that are used to induce cancer cell apoptosis function not only through the dysregulation of Ca^2+^ signaling but also via the activation of other apoptotic modulators^26,27^, such as death receptor- and/or mitochondria-dependent pathways^28,29^. Ca^2+^ is consdered to be a link between ER stress and mitochondrial apoptotic pathways^30,31^. SOCE, by definition, is activated by Ca^2+^ efflux from the internal store. Stromal-interaction molecule 1 (STIM1) is a Ca^2+^ sensor in the ER that triggers SOCE pathway activation. Once Ca^2+^ is depleted in the ER, STIM1 proteins aggregate into multiple puncta and translocate to regions with close proximity to the plasma membranes^32^. Activated STIM1 binds to Orai1 (CRACM1, calcium release-activated calcium modulator 1) or/and TRPC1 (transient receptor potential canonical 1), two Ca^2+^ channels, located in the plasma membrane, which allow Ca^2+^ entry and are therefore involved in SOCE^33–35^. TG, a sarco/endoplasmic reticulum Ca^2+^-ATPase (SERCA) inhibitor that induces SOCE drastically following the depletion of Ca^2+^ stores of the ER, has been shown to induce apoptosis^36^. However, the underlying mechanism of Bcl-2-mediated regulation of TG-induced apoptosis has not been clearly elucidated.

Interaction of Bcl-2 with Bax appears to be important for its activity, and the BH1 and BH2 domains of Bcl-2 are required for the inhibition of apoptosis and heterodimerization with Bax^37^. The α5-helix within the BH1 domain of Bcl-2 is essential for its cytoprotective function. Bcl-2, which lacks a BH1 domain (Bcl-2/ΔBH1) or bears alanine as a substitute for glycine 145 in the BH1 domain (Bcl-2/G145A), fails to interact with either Bax or Bak, accelerating Bax- or Bak-induced apoptosis. Bcl-2/ΔBH1 or Bcl-2/G145A acts as a dominant-negative mutation of endogenous anti-apoptotic proteins such as Bcl-2 and Bcl-xL^38^. Furthermore, Bcl-2, but not the inactive point mutant Bcl-2/G145A, undergoes a conformational change in response to apoptotic agonists^39^. Bcl-2/G145A completely abrogated the death-repressor activity of Bcl-2 and disrupted its heterodimerization with Bax. Therefore, the structure of this region of Bcl-2 is important for its biological function^40^. In this study, in order to focus specifically on the effect of Bcl-2 on Ca^2+^-mediated apoptosis, we chose the apoptosis-inducing agent TG as an ER stress inducer to disrupt Ca^2+^ homeostasis in the ER^41^. We observed that Bcl-2 downregulated the basal Ca^2+^ levels in cytosol and mitochondria and prohibited Ca^2+^ elevation under apoptotic stimulation. We also observed that SOCE contributed to the TG-induced Ca^2+^ elevation and cytotoxicity when the α5-helix of Bcl-2 was mutated.

## Results

### Both wild-type and Bcl-2 mutants are localized in the ER and mitochondria

**Fig. 1:**
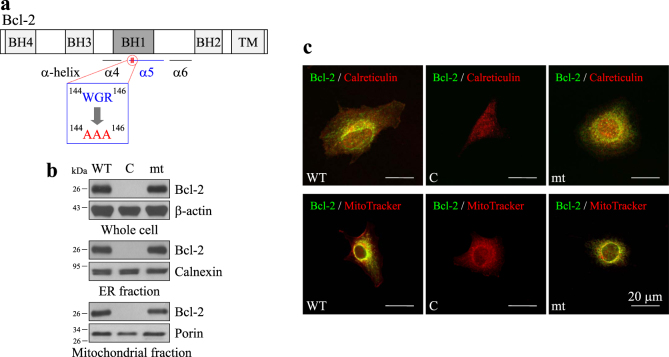
The expression and subcellular distribution of wild-type and mutant Bcl-2 in MDCK cells. **a** Point mutations of three-amino acids, ^144^WGR^146^ to ^144^AAA^146^, in the Bcl-2 α5-helix motif within the BH1 domain. **b** Western immunoblotting of Bcl-2, β-actin, calnexin, and porin. SDS-PAGE for the whole-cell lysate, ER lysate, and mitochondrial lysates from MDCK cells that overexpressed control vector (C), wild-type Bcl-2 (WT), and Bcl-2 mutant (mt). β-actin, calnexin, and porin were used as the internal control for whole-cell, ER, and mitochondrial lysates, respectively. **c** Immunofluorescence staining was performed to label Bcl-2, ER, and mitochondria, and the fluorescence images were obtained using confocal microscopy (scale bar, 20 μm). Calreticulin and MitoTracker Orange were used as the ER and mitochondrial markers, respectively

A three-amino-acid mutation (^144^WGR^146^ to ^144^AAA^146^) was generated within the α5-helix of the Bcl-2 BH1 domain (Fig. 1a). We established Bcl-2 overexpressing stable clones of endogenous Bcl-2-free cells using MDCK and two other cervical cancer cell lines (SiHa and HeLa). Organelle fragmentation for Western blotting (Fig. 1b) and immunofluorescence staining (Fig. 1c) was performed to prove that both wild-type Bcl-2 (WT) and the Bcl-2 mutant (mt) were localized in the ER and mitochondria. In addition, overexpression of the isopropyl-beta-D-thiogalactoside (IPTG)-inducible WT in MDCK cells exhibited similar distribution of non-inducible WT (Supplementary Fig. 1a).

### Mutation of Bcl-2 enhances TG-induced apoptosis

**Fig. 2:**
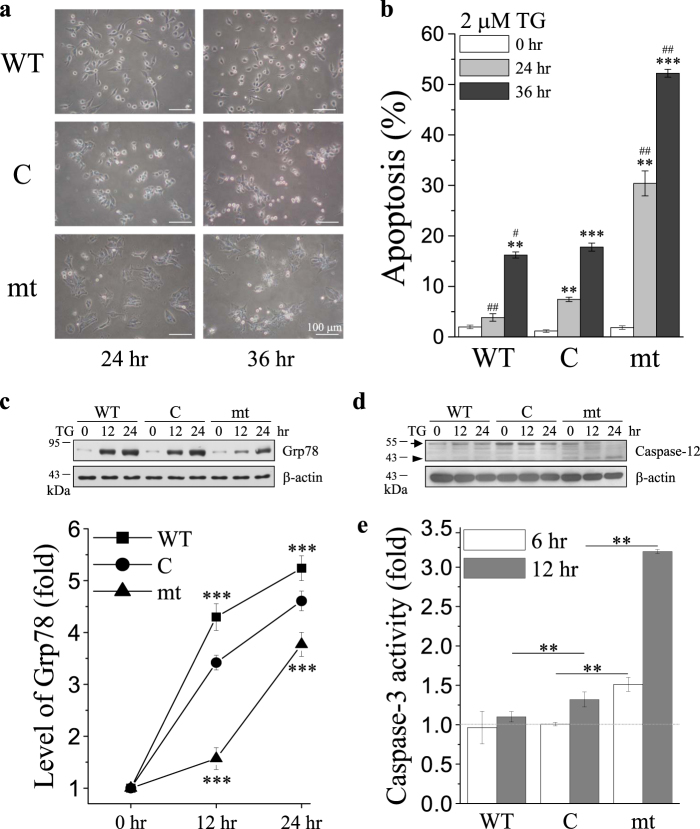
Bcl-2 inhibits thapsigragin (TG)-induced ER stress-mediated apoptosis. **a**, **b** Overexpression of control vector (C), wild-type Bcl-2 (WT), and Bcl-2 mutant (mt) in MDCK cells that were treated with 2 μM TG for 48 h and 72 h. **a** Representative images of cells as observed under a bright-field microscope (scale bar, 100 μm). **b** Quantitative analysis of the apoptosis ratio that was assessed from the hypodiploid DNA peak of propidium iodide (PI)-stained cells by flow cytometry from five independent experiments. The data were found to be statistically significant at *p* < 0.01 (indicated by **) and *p* < 0.001 (indicated by ***) compared to untreated cells (0 h) of the same cell line, and *p* < 0.05 (indicated by #) and *p* < 0.01 (indicated by ##) compared to control vector (C) overexpressing cells at the same time point (Student’s *t*-test). **c**,** d** Cells were treated with 2 μM TG, and the cell lysates were harvested at the indicated time points. The lysates were analyzed by SDS-PAGE and Western blotting for Grp78, caspase-12, and the internal control β-actin. Arrow and arrowhead indicate the inactive and active caspase-12, respectively. Representative pictures of three independent experiments. **c** Lower panel, densitometry was performed to quantify the relative level of Grp78 expression from three independent Western blots. The data were found to be statistically significant at *p* < 0.001 (indicated by ***) compared to control vector (C)-overexpressing cells at the same time point (Student’s *t*-test). **e** Caspase-3 activity was measured using the fluorogenic substrate DEVD-AFC cells treated with 2 μM TG for 6 h or 12 h. The data were found to be statistically significant at *p* < 0.01 (indicated by **) compared to control vector (C)-overexpressing cells at the same time point (Student’s *t*-test)

The cells that overexpressed the WT resisted TG-induced apoptosis, whereas cells that overexpressed mt enhanced TG-induced apoptosis (Fig. 2a, b). Furthermore, overexpression of IPTG-inducible WT decreased TG-induced apoptosis in a dose-dependent manner (Supplementary Fig. 1b). In contrast, overexpression of mt enhanced TG-induced apoptosis of cervical cancer cells in a time-dependent manner (Supplementary Fig. 1c, d). These results indicate that the Bcl-2 α5-helix can modulate TG-induced Ca^2+^ cytotoxicity.

### TG-induced ER stress-mediated apoptosis

**Fig. 3:**
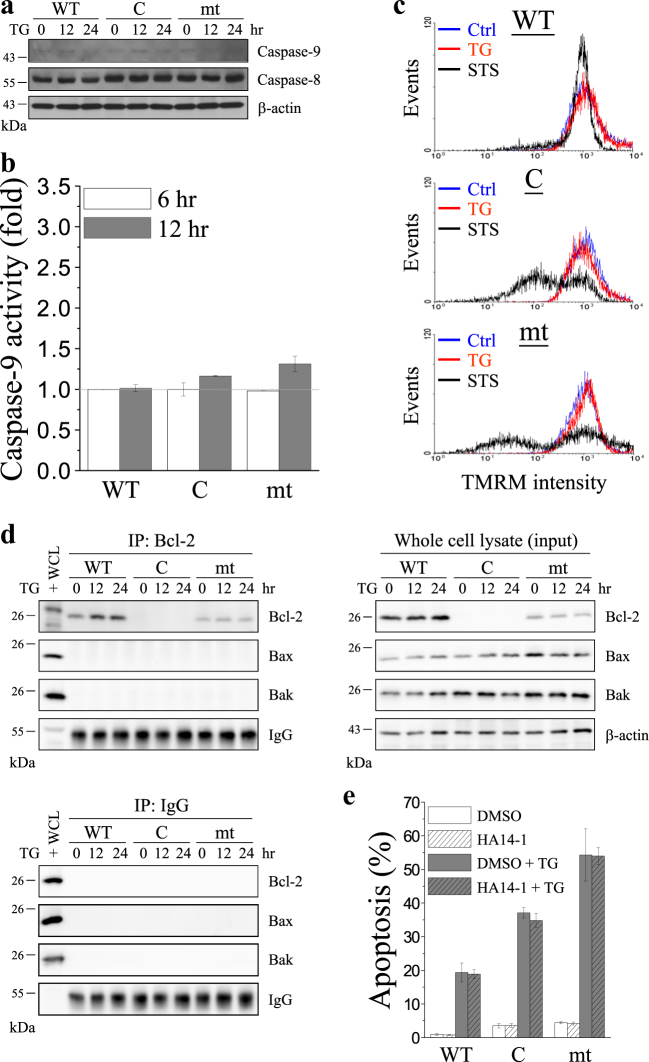
Death receptor- or mitochondria-dependent pathways, and the interaction of Bcl-2 with Bax are not involved in thapsigragin (TG)-induced apoptosis. **a** Control vector (C)-, wild-type Bcl-2 (WT)-, and Bcl-2 mutant (mt)-overexpressing MDCK cells were treated with 2 μM TG, and the cell lysates were harvested at the indicated time points. The lysates were analyzed by SDS-PAGE and Western blotting for caspase-9, caspase-8, and the internal control β-actin. Representative pictures of three independent experiments. **b** Caspase-9 activity was measured using the fluorogenic substrate LEHD-AFC in cells treated with 2 μM TG for 6 or 12 h. (*n* = 3; Student’s *t*-test). **c** Cells were treated with 2 μM TG or 500 nM staurosporine (STS) for 12 h and subsequently stained with 500 nM TMRM for 30 min. Mitochondrial membrane potential was analyzed based on the TMRM intensity observed by performing flow cytometry. STS-induced decrease of TMRM fluorescence was used as the positive control. The *x*-axis indicates TMRM fluorescence intensity, and the *y*-axis indicates cell number (*n* = 3; Student’s *t*-test). **d** Cells were treated with 2 μM TG for 12 or 24 h, and cell lysates were harvested at the indicated time points. The lysates were subjected to immunoprecipitation using anti-Bcl-2 antibody (upper-left) or an IgG isotype control (lower-left), followed by analysis of the precipitated proteins by SDS-PAGE and immunoblotting with Bcl-2, Bax, and Bak antibodies. The whole-cell lysate of wild-type Bcl-2 (WT)-overexpressing MDCK cells was used as positive control (indicated by + ). Western blotting of Bcl-2, Bax, Bak, and the internal control β-actin were performed in whole-cell lysates to represent input control (upper-right). **e** Cells were pre-incubated with or without 5 μM HA14-1 for 1 h and subsequently treated with or without 2 μM TG for 36 h. Quantitative analysis of the apoptosis ratio was assessed from the hypodiploid DNA peak of propidium iodide (PI)-stained cells from three independent experiments by flow cytometry (Student’s *t*-test)

Overexpression of glucose-regulated protein 78 (Grp78), an ER-resident chaperone protein and a hallmark of ER stress, can prevent cell death by protecting against many apoptotic stimulations. The results revealed that after TG treatment, Grp78 was upregulated in the cells that overexpressed WT, and downregulated in the cells that overexpressed mt to that in MDCK cells (C) (Fig. 2c). Cleavage of procaspase-12, procaspase-8, procaspase-9, and procaspase-3 are indicators of activation of ER stress-, death receptor-, mitochondria-dependent, or common apoptotic pathway, respectively. Herein, cleavage of procaspase-12 and induction of the ER stress, which specifically activated caspase-12, were found in cells that overexpressed mt, whereas overexpression of WT inhibited the activation of caspase-12 (Fig. 2d) and the effector caspase-3 (Fig. 2e). Neither WT nor mt activated caspase-8 (Fig. 3a) or caspase-9 (Fig. 3b) or caused the loss of mitochondrial membrane potential (Fig. 3c) after TG treatment. These results implied that ER stress, but not mitochondria-dependent or death receptor-dependent pathways, contributed to the TG-induced apoptosis.

### Bcl-2 and Bax interaction is not involved in TG-induced apoptosis

**Fig. 4:**
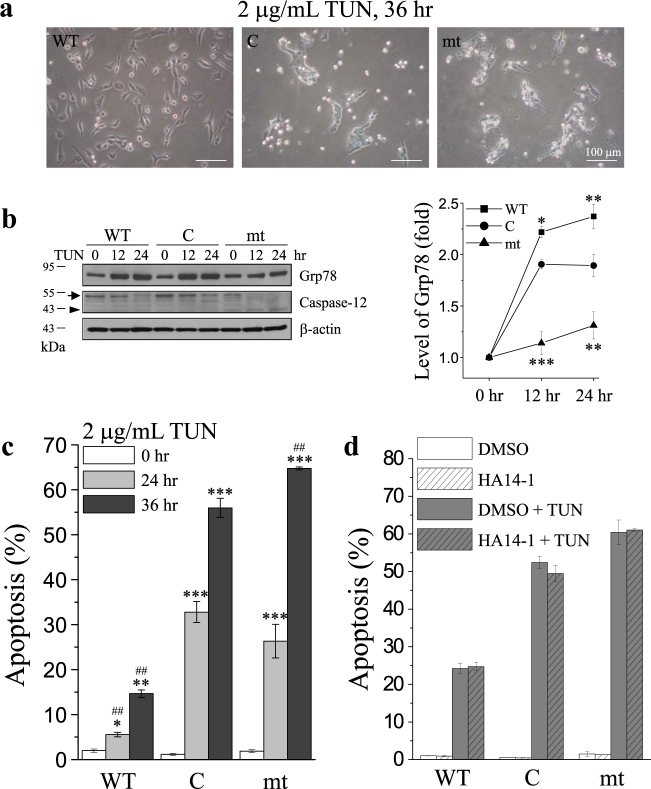
Bcl-2 inhibits tunicamycin (TUN)-induced ER stress-mediaed apoptosis. Control vector (C)-, wild-type Bcl-2 (WT)-, and Bcl-2 mutant (mt)-overexpressing MDCK cells were treated with 2 μg/ml TUN for 12, 24, or 36 h. **a** Representative images of cells were taken under a bright-field microscope (scale bar, 100 μm). **b** Cells were treated with 2 μg/ml TUN, and cell lysates were harvested at the indicated time points. The lysates were analyzed by SDS-PAGE and Western blotting for Grp78, caspase-12, and the internal control β-actin. Arrow and arrowhead indicate the inactive and active caspase-12, respectively. Representative pictures of three independent experiments. **c** Quantitative analysis of the apoptosis ratio was assessed from the hypodiploid DNA peak of propidium iodide (PI)-stained cells from five independent experiments by flow cytometry. The data were found to be statistically significant at *p* < 0.05 (indicated by *), *p* < 0.01 (indicated by **), and *p* < 0.001 (indicated by ***) compared to untreated cells (0 h) of the same cell line, and *p* < 0.01 (indicated by ##) compared to control vector (C)-overexpressing cells at the same time point (Student’s *t*-test). **d** Cells were pre-incubated with or without 5 μM HA14-1 for 1 h and subsequently treated with or without 2 μg/ml TUN for 36 h. Quantitative analysis of the apoptosis ratio was assessed from the hypodiploid DNA peak of propidium iodide (PI)-stained cells from three independent experiments by flow cytometry (Student’s *t*-test)

Immunoprecipitation results revealed no interaction between Bcl-2 and Bax under TG treatment, regardless of the overexpression of WT or mt (Fig. 3d). Conversely, HA14-1, an inhibitor of Bcl-2, prevents Bcl-2 from interacting with Bax, thereby inhibiting the anti-apoptotic effect of Bcl-2 and sensitizes cells to induction of apoptosis. As shown in this study, cells that overexpressed WT or mt, HA14-1 did not change the level of TG-induced apoptosis (Fig. 3e). In addition, we also tested the effect of Bcl-2 on tunicamycin (TUN)-induced apoptosis. TUN, a nucleoside antibiotic and an ER stress inducer, inhibits protein glycosylation, resulting in the accumulation of proteins in the ER and causing ER stress. TUN has been reported to increase cytosolic Ca^2+^ levels by Ca^2+^ efflux from the Ca^2+^ pool in the ER, and influx of extracellular Ca^2+^ across the plasma membrane^42^. As shown in Fig. 4, mt enhanced TUN-induced caspase-12 activation and apoptosis (Fig. 4a–c), but downregulated Grp78 (Fig. 4b). In contrast, WT exerted opposite effects on TUN-induced cell death, compared to mt. Similar to the results observed for TG-induced apoptosis treatment, inhibition of the interaction of Bcl-2 with Bax by HA14-1 did not affect TUN-induced apoptosis (Fig. 4d).

### Bcl-2 α5-helix regulates intracellular Ca^2+^ compartmentation

**Fig. 5:**
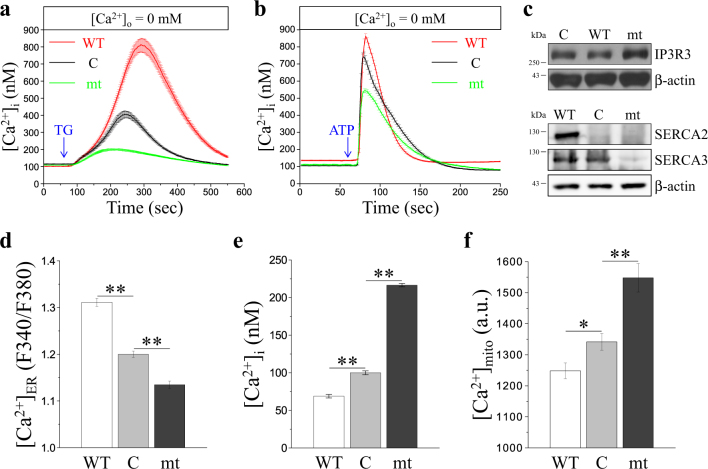
The putative Bcl-2 pore-forming domain regulates intracellular Ca^2+^ compartmentation. **a**,** b** Cells were loaded with 2 μM fura-2/AM at 37 °C for 30 min prior to cytosolic Ca^2+^ measurement. Representative curves for the measurement of ER-releasable Ca^2+^ are represented as mean ± SEM (where, *n* ≥ 80 cells) from three independent experiments in control vector (C)-, wild-type Bcl-2 (WT)-, and Bcl-2 mutant (mt)-overexpressing MDCK cells. An arrow indicates that cells were stimulated with 2 μM thapsigargin (TG) or 100 μM ATP at 1 min to extrude ER lumen-resident Ca^2+^ in Ca^2+^-free buffer as ER-releasable Ca^2+^. **c** Western immunoblotting of IP3R3, SERCA2, SERCA3, and the internal control β-actin was performed in Bcl-2 overexpressing MDCK cells. A representative of three independent experiments. The cells were loaded with **d** 2 μM mag-fura-2/AM, **e** 2 μM fura-2/AM, or **f** 2 μM rhod-2/AM at 37 °C for 30 min to fluorescently indicate the ER Ca^2+^ ([Ca^2+^]_ER_), cytosolic Ca^2+^ ([Ca^2+^]_i_), and mitochondrial Ca^2+^ ([Ca^2+^]_mito_), respectively. Quantitative analysis of the relative Ca^2+^ levels was performed by measurement of fluorescence intensity of these Ca^2+^ indicators. All values are represented as mean ± SEM (where, *n* ≥ 150 cells). The data were found to be statistically significant at *p* < 0.05 (indicated by *) and *p* < 0.01 (indicated by **) compared to control vector (C)-overexpressing cells (Student’s *t*-test)

Depletion or overload of Ca^2+^ in cytosol or organelles may cause stress and Ca^2+^ cytotoxicity, leading to cell death. At first, we evaluated Ca^2+^ levels in the Ca^2+^ pool of ER stores by passive TG-induced Ca^2+^ leakage and active ATP-induced Ca^2+^ release from the ER into the cytosol. We found that overexpression of WT and mt increased and decreased the Ca^2+^ concentration within the ER, respectively (Fig. 5a, b). IP3Rs and SERCAs are major molecules acting in response to the release of Ca^2+^ from the ER and uptake of Ca^2+^ into the ER, respectively. As shown in Fig. 5c, overexpression of WT downregulated IP3R3 and upregulated SERCA2 and SERCA3. In contrast, overexpression of mt upregulated IP3R3 and downregulated SERCA3 (Fig. 5c). Additionally, organelle-specific fluorescent Ca^2+^ indicators, mag-fura-2/acetoxymethyl ester (mag-fura-2/AM), fura-2/acetoxymethyl ester (fura-2/AM), and rhod-2/acetoxymethyl ester (rhod-2/AM), were used to identify ER ([Ca^2+^]_ER_), cytosolic ([Ca^2+^]_i_), and mitochondrial ([Ca^2+^]_mito_) Ca^2+^ levels in the resting state, respectively. Cells that overexpressed mt showed a lower Ca^2+^ level in the ER but a higher Ca^2+^ level in the cytosol and mitochondria (Supplementary Fig. S2 and Fig. 5d–f). However, cells that overexpressed WT showed a higher Ca^2+^ level in the ER but lower Ca^2+^ level in the cytosol and mitochondria. High cytosolic Ca^2+^ concentration can activate calpains, which can regulate cellular functions and cause cell death by proteolyzing its cellular substrates. The fluorogenic substrate, *t*-Boc-LM-CMAC, was used to evaluate the basal activity of calpains. Our data showed that WT decreased calpain activity, whereas mt increased calpain activity (Fig. S3a, b). In addition, changes in the levels of μ-calpain expression also corresponded with the calpain activity as shown by cleavage of the calpain substrate, α-spectrin (Fig. S3c).

### Bcl-2 α5-helix is important in regulating Ca^2+^ homeostasis

**Fig. 6:**
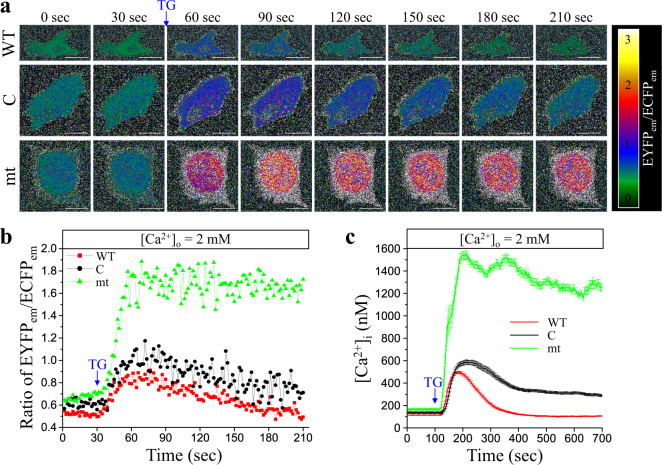
The putative Bcl-2 pore-forming domain regulates thapsigargin (TG)-induced Ca^2+^ elevations. **a**, **b** Monitoring changes in cytosolic Ca^2+^ level by cameleon, a FRET-based Ca^2+^ indicator, expressed in control vector (C)-, wild-type Bcl-2 (WT)-, and Bcl-2 mutant (mt)-overexpressing MDCK cells. The relative emission ratios (EYFP_em_/ECFP_em_) from EYFP and ECFP under excitation of ECFP were measured in 2 mM Ca^2+^ buffer every 1.5 s for 140 frames using confocal microscopy. TG was added at the time point of 30 s during time-lapse recording. **a** Representative ratio images of EYFP_em_/ECFP_em_ were shown every 30 s and presented in pseudocolor (scale bars, 20 μm). **b** Quantitative analysis of the relative emission ratios (EYFP_em_/ECFP_em_) before and after TG treatment. Each curve represents the mean values from at least 10 cells. **c** Ratiometric dye, fura-2/AM, was used as a probe for cytosolic Ca^2+^ measurement using a single cell fluorimeter. Cells were loaded with 2 μM fura-2/AM at 37 °C for 30 min before TG stimulation. The arrow indicates that cells were stimulated with 2 µM TG at 100 s in 2 mM Ca^2+^ buffer. Representative curves data are represented as mean ± SEM (where, *n* ≥ 60 cells) from three independent experiments

TG-mediated Ca^2+^-ATPase inhibition allows Ca^2+^ to flow from the ER lumen into the cytoplasm, producing a transient elevation of cytosolic Ca^2+^ level, and triggering a sustained elevation of cytosolic Ca^2+^ level through SOCE. The genetically encoded FRET-based fluorescent protein, cameleon, and the ratiometric chemical dye, fura-2/AM, were used as indicators in Ca^2+^ measurement (Fig. 6). The ratio images (Fig. 6a) and ratio values (Fig. 6b) demonstrated that during TG treatment, overexpression of WT was negatively correlated with cytosolic Ca^2+^ elevation, whereas overexpression of mt was positively correlated with cytosolic Ca^2+^ elevation during TG treatment (Supplementary Video S1, S2). The cytosolic Ca^2+^ level in TG-treated cells that overexpressed WT returned to the original level rapidly. In contrast, cells that overexpressed mt showed dramatic elevation in the cytosolic Ca^2+^ level, and sustained this level for a long period of time. In addition, similar results were obtained with the ratiometric Ca^2+^ probe, fura-2/AM (Fig. 6c).

### Bcl-2 α-helix regulates SOCE pathway activation during TG treatment

**Fig. 7:**
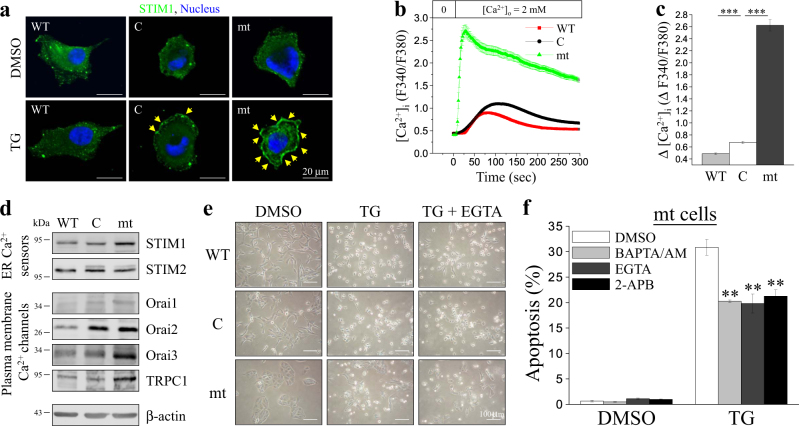
Bcl-2 harboring the α5-helix mutation disrupts of SOCE that contributed to thapsigargin (TG)-induced apoptosis. **a** Control vector (C)-, wild-type Bcl-2 (WT)-, and Bcl-2 mutant (mt)-overexpressing MDCK cells were treated with DMSO or TG for 5 min. Subsequently, immunofluorescence staining was obtained to label STIM1 and nucleus. Representative fluorescence images were obtained using confocal microscopy (scale bar, 20 μm). Arrows indicate the translocation of STIM1 to the juxta-plasma membrane region. **b**, **c** Pre-incubation of cells with 2 μM fura-2/AM at 37 °C for 30 min for cytosolic Ca^2+^ measurement using a single cell fluorimeter. Depletion of ER lumen-resident Ca^2+^ was induced by treating cells in Ca^2+^-free buffer with 2 μM TG for 10 min. The subsequent elevation of Ca^2+^ indicated that SOCE occurred during the exchange of Ca^2+^-free buffer to 2 mM-Ca^2+^ buffer. **b** The data in representative curves for the measurement of SOCE are represented as mean ± SEM (where, *n* ≥ 60 cells) from three independent experiments. **c** Quantitative analysis of the changes in the peak Ca^2+^ levels. All values are represented as mean ± SEM, and the data were found to be statistically significant at *p* < 0.001 (indicated by ***) compared to control vector (C)-overexpressing cells (Student’s *t*-test). **d** Western blotting of SOCE related molecules, such as ER Ca^2+^ sensors (STIM1 and STIM2), plasma membrane Ca^2+^ channels (Orai1, Orai2, Orai3, and TRPC1), and the internal control β-actin. **e** Cells were pre-incubated with 2 mM EGTA for 30 min and treated with DMSO or 2 μM TG for 24 h. Representative images of cells were obtained under a bright-field microscope (scale bar, 100 μm). **f** Bcl-2 mutant (mt)-overexpressing cells were pre-incubated with 20 μM BAPTA/AM, 2 mM EGTA, or 2 μM 2-APB for 30 min and treated with DMSO or 2 μM TG for 24 h. Quantitative analysis of the apoptosis ratio was assessed from the hypodiploid DNA peak of propidium iodide (PI)-stained cells from three independent experiments by flow cytometry. The data were found to be statistically significant at *p* < 0.01 (indicated by **) compared to the DMSO control (Student’s *t*-test)

We found that mt highly enhanced and sustained the cytosolic Ca^2+^ elevation during TG treatment. Therefore, we investigated whether Bcl-2 can regulate STIM1 translocation to plasma membrane and cause SOCE pathway activation. Confocal microscopy images showed that WT reduced STIM1, which acts as Ca^2+^ sensors in the ER, that led to their translocation to the juxta-plasma membrane regions during TG-induced Ca^2+^ depletion. In contrast, mt significantly promoted the aggregation of most of the STIM1 proteins into multiple puncta and translocated them to the juxta-plasma membrane region (Fig. 7a). As shown in Fig. 7b, c, the increase in SOCE activation, as observed by Ca^2+^ measurement, is consistent with the translocation ability of STIM1 shown in Fig. 7a. In addition to SOCE pathway activation, the expression of SOCE-related molecules was examined. Western blotting results also demonstrated that WT decreased the level of STIM1, Orai1, and Orai2, resulting in reduction of SOCE. In contrast, Bcl-2 increased the level of STIM1, Orai1, Orai2, Orai3, and TRPC1 expression (Fig. 7d), leading to the enhancement of SOCE and Ca^2+^ burst. The extracellular Ca^2+^ chelator, EGTA, inhibited TG-induced apoptosis significantly (Fig. 7e). Furthermore, TG-induced apoptosis of the cells overexpressing mt could be reduced by intracellular Ca^2+^ chelator, BAPTA/AM, extracellular Ca^2+^ chelator, EGTA, and the SOCE inhibitor, 2-APB (Fig. 7f).

## Discussion

Ca^2+^ is an important secondary messenger that regulates multiple cellular processes^21^. Bcl-2 exerts a direct effect on the Ca^2+^ handling in the ER by regulating the movement of Ca^2+^ through the ER membrane^14,25,43^. However, the effect of Bcl-2 on the intracellular and intra-organellar Ca^2+^ level remains controversial as it is supported by conflicting data. Overexpression of Bcl-2 in human breast epithelial cells and mouse lymphoma cells increases [Ca^2+^]_ER_^44,45^. In contrast, Bcl-2 overexpression in human prostate cancer cell^46^, HEK-293 cells, and R6 fibroblasts^17^ decreases [Ca^2+^]_ER_. Therefore, Bcl-2 appears to regulate [Ca^2+^]_ER_ through multiple mechanisms, and in a cell-type-specific manner. In this study, we demonstrated that WT increased [Ca^2+^]_ER_ but decreased [Ca^2+^]_i_ (Supplementary Fig. S2a, b, and Fig. 5), which may be due to increased Ca^2+^ uptake into the ER or decreased Ca^2+^ leakage from the ER. In contrast, mt decreased [Ca^2+^]_ER_ and increased [Ca^2+^]_i_ (Supplementary Fig. S2a, b, and Fig. 5), which may be due to decreased Ca^2+^ uptake into the ER or increased Ca^2+^ leakage from the ER. In addition, the mitochondrial Ca^2+^ level appeared to be responding to the cytosolic Ca^2+^ level (Supplementary Fig. S2c and Fig. 5f). Consequently, the α5-helix of Bcl-2 is involved in Bcl-2-mediated Ca^2+^ distribution. These phenomena could be explained by the changes in the level of IP3R3, SERCA2, and SERCA3, or disrupted structural or functional interaction between Bcl-2 and IP3Rs or SERCAs (Fig. 5c).

In this study, we found that overexpression of mt enhanced TG-induced apoptosis which selectively activated ER-stress-related apoptosis. This conclusion was supported by the following findings: (i) Most cells that overexpressed mt rounded up with the appearance of apoptotic bodies after TG treatment (Fig. 2a), which was confirmed by propidium iodide (PI) staining (Fig. 2b and Supplementary Fig. S1). (ii) Several ER-stress-related molecules were activated and procaspase-12 was cleaved into the active caspase-12 fragment, suggesting the activation of caspase-12 (Fig. 2d); procaspase-3, the downstream target of caspase-12, was successively activated (Fig. 2e); and Grp78, the ER chaperone and ER stress responder, was overexpressed after TG treatment. (iii) The death receptor- and mitochondria-dependent signal pathways were not involved in TG-induced apoptosis because there were no significant changes in caspase-8 and caspase-9 activation and in the mitochondrial membrane potential (Fig. 3). Previous studies show that Grp78 prevents apoptosis induced by the disruption of Ca^2+^ homeostasis in the ER^47^. Conversely, suppression of Grp78 expression causes an increase in cell death induced by Ca^2+^ depletion in the ER^48^. In the present study, after treatment with ER stress inducer (TG and TUN), the level of Grp78 expression in the cells that overexpressed WT was higher than that in cells that overexpressed mt (Fig. 2c and Fig. 4b). Our data indicated the effect of the Bcl-2 α5-helix on Grp78 expression in relation to the regulation of ER stress-induced apoptosis. Furthermore, intracellular Ca^2+^ chelator (BAPTA-AM), extracellular Ca^2+^ chelator (EGTA), and SOCE inhibitor (2-APB) attenuated TG-induced apoptosis (Fig. 7f). These results indicated that mt depleted the intracellular Ca^2+^ stores to enhance the ER stress-induced apoptosis which involved SOCE-mediated sustained elevation of intracellular Ca^2+^.

The BH1 and BH2 domains of Bcl-2 are required for inhibition of apoptosis and heterodimerization with Bax^37^. Therefore, mutation of the Bcl-2 α5-helix within the BH1 domain may disrupt the interaction between Bcl-2 and Bax. Herein, overexpression of WT led to the downregulation of the pro-apoptotic Bcl-2 proteins, Bax and Bak, and increased the anti-apoptotic Bcl-2 activity, which was confirmed using anti-active Bcl-2 antibody. In contrast, overexpression of mt increased Bax activity, which was confirmed using anti-active Bax antibody (Fig. S4). Immunoprecipitation and HA14-1 pretreatment showed that the interaction between Bcl-2 and Bax is not involved in TG and TUN-induced apoptosis (Figs. 3 and 4).

SOCE helps to replenish the Ca^2+^ pool in the ER, where the filling state of the intracellular Ca^2+^ stores regulates the entry of Ca^2+^ across the plasma membrane. We found that overexpression of WT increased [Ca^2+^]_ER_ and slightly decreased SOCE. The slight decrease in SOCE could be a direct consequence of WT overexpression or an adaptive mechanism in response to the long-term elevation of Ca^2+^ content in the ER^49^. Additionally, we observed a decrease in [Ca^2+^]_ER_ and significant increase in SOCE in cells that overexpressed mt (Fig. 5a, b, and Fig. 7b). In principle, a reduction in the steady state [Ca^2+^]_ER_ level due to mt overexpression should activate SOCE. It is indicated that overexpression of mt placed the cells at a [Ca^2+^]_ER_-depleted status. TG-induced [Ca^2+^]_i_ increase via emptying of the Ca^2+^ stores in the ER and activation of SOCE pathway has been shown to induce apoptosis in a wide variety of cell types^21,42,50^. TG-mediated Ca^2+^-ATPase inhibition allows Ca^2+^ transport from the ER lumen into the cytoplasm, leading to a transient elevation of cytosolic Ca^2+^ concentration, followed by sustained elevation of cytosolic Ca^2+^ due to SOCE^51^. The results of this study showed that overexpression of WT could reduce sustained elevation of cytosolic Ca^2+^ induced by TG, and nullify the Ca^2+^ disturbance rapidly (Fig. 6). However, overexpression of mt helped to maintain the sustained elevation of cytosolic Ca^2+^ (Fig. 6), leading to ER stress and cell apoptosis (Fig. 2 and Supplementary Fig. S1).

**Fig. 8:**
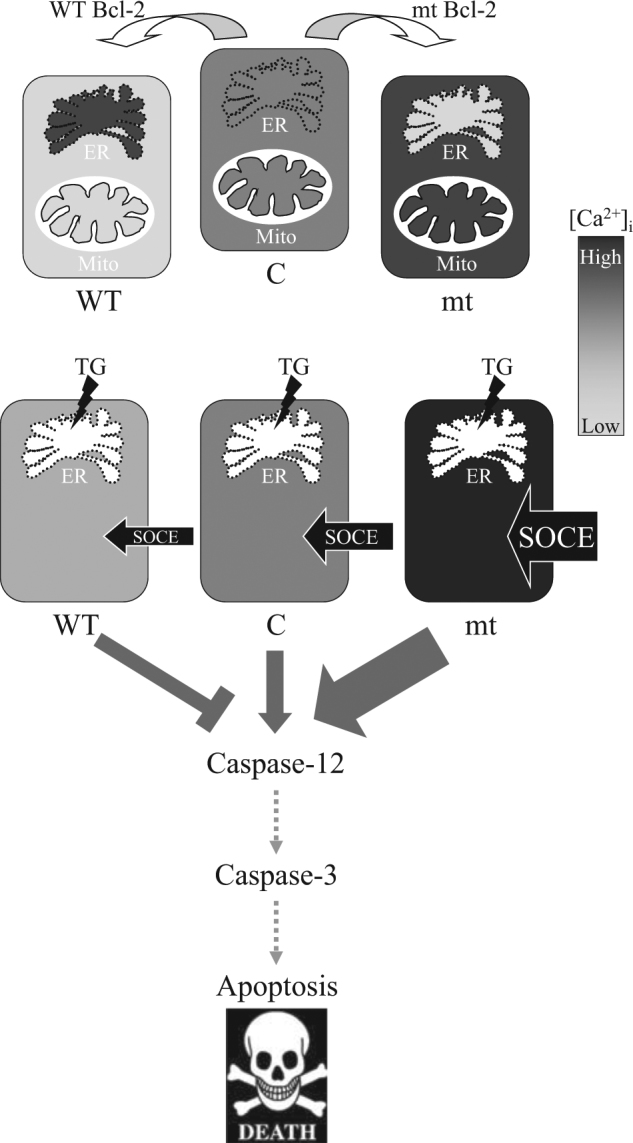
Schematic diagram of Ca^2+^ homeostasis regulation by Bcl-2. Wild-type Bcl-2 (WT) maintains high Ca^2+^ levels in the ER but low Ca^2+^ levels in cytosol and mitochondria. The three-amino acid mutation (^144^WGR^146^ to ^144^AAA^146^) in the Bcl-2 α5-helix (mutant Bcl-2, mt) depletes Ca^2+^ in the ER store but causes Ca^2+^overload in both cytosol and mitochondria. WT and mt cause different expression of SOCE-related molecules. During thapsigargin stimulation, mt-overexpressing cells presented a significant Ca^2+^ influx through SOCE and subsequent activation of ER-stress-mediated caspase activation and apoptosis. In contrast, WT-overexpressing cells decreases thapsigargin-induced SOCE and intracellular Ca^2+^ elevation which protects cells against apoptosis

As the Ca^2+^-ATPase SERCA is responsible for the Ca^2+^ uptake in the ER, it is possible that this protein is modulated by Bcl-2. In fact, increasing direct interaction between Bcl-2 and SERCA is associated with Bcl-2 expression, which has been demonstrated by immunoprecipitation studies^44^. Recently, strong experimental evidence indicated that Bcl-2 regulates the phosphorylation of IP3R, which in turn regulates the leak rate through the channel^52,53^. Another study showed that Bcl-2 reduces the probability opening of IP3Rs in the lipid bilayers^19^. Further studies are required to clarify the relationship between the Bcl-2 BH1 domain and other Ca^2+^-regulating molecules, such as IP3R3, SERCA2, or SERCA3. In conclusion, these results highlight the importance of Ca^2+^ regulation by the Bcl-2 BH1 domain in response to ER stress-mediated apoptosis. Bcl-2 with an α5-helix mutation was involved in SOCE pathway activation, which may be the cause of TG-induced apoptosis. In contrast, WT showed an anti-apoptotic effect by reducing SOCE pathway activation during TG-induced Ca^2+^ disturbance (Fig. 8).

## Materials and methods

### Cells and cell culture

MDCK, a cell line developed from the distal nephron of a dog, and human cervical carcinoma cell lines, SiHa and HeLa, were used in this study. The stable transfections were as follows: (i) overexpression of the WT gene; (ii) overexpression of the control vector (C); and (iii) overexpression of the mt gene. These cells were maintained in DMEM supplemented with 10% FBS under 5% CO_2_ at 37 °C.

### The DNA constructs and transfection

The vector pCΔj contained G418 resistance gene and EBV-derived replication origin. The human *bcl-2* cDNA sequences inserted into pCΔj vector (pCΔj-bcl-2) were expressed by simian virus 40 (SV40) enhancer/promoter regulatory elements^54^. A DNA construct without *bcl-2* cDNA sequences (pCAj-SV2) was used as a control. Transfection of cells was done using Lipofectamine™ 3000 Reagent (Thermo Fisher Scientific, Waltham, MA). Foty-eight hours after transfection, cells were passaged by 1:10 dilution into G418 (500 μg/ml) selective medium. The IPTG-inducible expression of Bcl-2 by the lac operator/repressor system was established^55^. The stable transfection of a constitutively expressed lacI gene, encoding lac repressor, and the human *bcl-2* gene that had been inserted downstream of a SV40 promoter containing the lac operator sequence in MDCK cells. The expression of the bcl-2 gene could be specifically activated by administration of the lactose analog IPTG.

### Western blotting

Cell lysates were harvested in RIPA buffer (150 mM NaCl, 1 mM EGTA, 50 mM Tris at pH 7.4, 10% glycerol, 1% Triton X-100, 1% sodium deoxycholate, 0.1% SDS, and Complete^TM^), and the lysates were analyzed by Western blotting using antibodies against Bcl-2 (DAKO, Grostrup, Denmark), Bax, Bak, calnexin, SERCA2, β-actin (Santa Cruz Biotechnology, Santa Cruz, CA), porin (Molecular Probes, Eugene, OR), caspase-8, caspase-9, caspase-12, SERCA3, pSer70-Bcl-2, pThr167-Bax (Cell Signaling Technology, Beverly, MA), Grp78, STIM1, STIM2, IP3R3 (BD, Franklin Lakes, NJ), Orai1, Orai2, Orai3, and TRPC1 (ProSci, Poway, CA).

### Fractionation of organelles

Cell homogenates were obtained using Dounce homogenizer along with a non-detergent lysis buffer (50 mM Tris-HCl at pH 7.4, 4 mM ETDA, 2 mM EGTA, 20 μg/ml leupeptine, 50 μM PMSF) on ice. The homogenate was centrifuged at a low-speed of 1000×*g* for 10 min, following which the supernatant was subjected to a medium-speed centrifugation at 20,000×*g* for 30 min. The pellets, which contained the mitochondria, were dissolved using 1% Triton X-100 lysis buffer. Meanwhile, the supernatant from the medium-speed centrifugation step was subjected to high-speed centrifugation at 40,000×*g* for 60 min, and the pellets containing the ER were dissolved using 1% Triton X-100 lysis buffer.

### Flow cytometric analysis

Cells were stained with 500 nM tetramethyl rhodamine methyl ester (TMRM; Molecular Probes, Eugene, OR), an indicator of mitochondrial membrane potential. The TMRM-stained cells were incubated in the dark at room temperature for 30 min and subsequently analyzed by flow cytometry (BD FACSCalibur, San Jose, CA) with excitation wavelength at 543 nm. In order to analyze the apoptotic ratio, the cells were fixed in 70% alcohol, followed by treatment with RNAse (100 mg/ml), and stained with PI (40 mg/ml) (Sigma, Saint Louis, MO). The PI-stained cells were incubated in the dark at room temperature for 30 min and analyzed by flow cytometry (BD FACSCalibur, San Jose, CA) with excitation wavelength at 543 nm. The apoptotic ratio was assessed from the hypodiploid DNA peak of apoptotic cells (sub G0/G1 phase) using Cell Quest software.

### Intracellular Ca^2+^ measurement

Cytosolic Ca^2+^ was measured at 37 °C using the fura-2 fluorescence ratio method on a single cell fluorimeter. Cells were loaded with 2 μM fura-2/AM in DMEM culture medium at 37 °C for 30 min. The excitation wavelength was alternated between 340 nm (*I*_340_) and 380 nm (*I*_380_) using the Polychrome IV monochromator (Till Photonics, Grafelfing, Germany). The fluorescence intensity was monitored at 510 nm, stored digitally, and analyzed by the program of TILLvisION 4.0 (Till Photonics).

### Evaluation of organellar Ca^2+^ levels

The cells were loaded with 2 μM fura-2/AM, 2 μM mag-fura-2/AM, or 2 μM rhod-2/AM, a direct probe for the cytosolic, ER, or mitochondrial Ca^2+^, in DMEM culture medium at 37 °C for 30 min. Fluorescence intensity of fura-2/AM and mag-fura-2/AM were observed under a single cell fluorimeter (Till Photonics, Grafelfing, Germany) and presented with pseudocolor images. Fluorescence images of rhod-2/AM were captured using a confocal imaging system (Olympus FV-1000, Tokyo, Japan).

### Evaluation of cytosolic Ca^2+^ levels by the fluorescent protein cameleon

We performed the stable transfection of the cells with the cameleon plasmid, followed by live-cell imaging using a confocal imaging system (Olympus FV-1000, Tokyo, Japan). An optional 440 nm LD laser was used to directly excite the enhanced cyan fluorescent protein (ECFP) without exciting the enhanced yellow fluorescent protein (EYFP). Conversely, emission spectra of ECFP and EYFP were 470–500 and 535–565 nm, respectively. The analysis program in FV-1000 imaging system allowed ratio calculation and ratio image acquisition after subtracting the background fluorescence intensity.

### Immunofluorescence staining and imaging

Cells were fixed with 4% buffered paraformaldehyde, permeabilized using 0.5% Triton X-100 for 15 min, and stained with mouse anti-Bcl-2 antibody (DAKO, Grostrup, Denmark), mouse anti-STIM1 antibody (BD, Franklin Lakes, NJ), or rabbit anti-calreticulin antibody (Upstate, Charlottesville, VA) for 12 h at 4 °C. In addition, cells were stained with goat anti-mouse IgG conjugated with Alexa 488 or goat anti-rabbit IgG conjugated with Alexa 594 (Molecular Probes, Eugene, OR) for 1 h. The fluorophore was excited by laser at 488 or 543 nm, respectively, and detected using a scanning confocal microscope (Olympus FV1000, Tokyo, Japan).

### Measurement of μ-calpain activity

The μ-calpain activity was assessed by generation of the fluorescent product, 7-amino-4-methoxy coumarin (AMC), from hydrolysis of an artificial μ-calpain fluorescent substrate *t*-Boc-LM-CMAC (Molecular Probes, Eugene, OR)^56^. Cells were pretreated with 10 μM *t*-Boc-LM-CMAC for 30 min. Intracellular fluorescence was sequentially imaged under a confocal imaging system (Olympus FV1000, Tokyo, Japan) and the mean fluorescence intensity (excitation by 405 nm LD laser) of individual cells was quantitatively analyzed.

### Quantitative analysis of caspase enzymatic activity

Activation of caspase-3 and caspase-9 was measured by a fluorimetric assay based on the specific hydrolysis of DEVD-AFC and LEHD-AFC (MBL, Nagoya, Japan), respectively. The cells were resuspended in chilled cell-lysis buffer kept on ice for 10 min followed by the addition of reaction buffer. Finally, 50 μM of DEVD- and LEHD-AFC was added, and the mixture was incubated at 37 °C for 2 h. The cleavage of DEVD- and LEHD-AFC was analyzed by measuring the release of AFC from DEVD- or LEHD-containing peptides. Fluorescence emission of AFC (excitation by 405 nm LD laser) was measured at 505 nm using a Fluoroskan AsCent FL fluorometer (Thermo Electron Corporation, Waltham, MA).

### Statistics

All data were represented as mean ± SEM (standard error of the mean) and Student’s *t*-test was performed for statistical analysis. A *p* value < 0.05 was considered significantly different.

## Electronic supplementary material

Supplementary information

Supplementary Video S1

Supplementary Video S2
