# Supplementary material for: Bcl-2 regulates store-operated Ca2+ entry to modulate ER stress-induced apoptosis
Source: Cell Death Discov. 2018 Feb 26;4:37. doi: 10.1038/s41420-018-0039-4 (PMC5841437; doi:10.1038/s41420-018-0039-4)
Supplement: Supplementary file 1 — Supplementary information [file 41420_2018_39_MOESM1_ESM.pdf]

Supplementary figure legends

Supplementary Figure S1. The Bcl-2 α5-helix modulates thapsigargin-induced apoptosis. (**a**) MDCK cells overexpressing the stably transfected IPTG-inducible human WT gene were pre-incubated with 10 μM IPTG for 24, 48, and 72 h, and lysates were collected from the whole-cell, ER, and mitochondrial fraction. Western blotting of Bcl-2, the internal control β-actin, ER marker calnexin, and mitochondrial marker porin was performed in MDCK cells that overexpressed Bcl-2. The wild-type Bcl-2 (WT)-overexpressing MDCK cells was used as positive control (indicated by +). (**b**) MDCK cells that overexpressed IPTG-inducible Bcl-2 after treatment with 10 μM IPTG for 0, 24, and 72 h and replacement of medium with DMSO or 2 μM TG for another 48 or 72 h of incubation. (**c** and **d**) Control vector (C)- or Bcl-2 mutant (mt)-overexpressing SiHa and HeLa cells were treated with 2 μM TG for 48 and 72 h. Level of Bcl-2 expression and that of the internal control β-actin was obtained by Western blotting. The wild-type Bcl-2 (WT)-overexpressing MDCK cells was used as positive control (indicated by +). Quantitative analysis of the apoptosis ratio was assessed from the hypodiploid DNA peak of propidium iodide (PI)-stained cells compared to the non-IPTG incubated cells (**b**) or vector control cells (**c** and **d**) by flow cytometry. All values are represented as mean ± SEM from three independent experiments. The data were found to be statistically significant at $p < 0.01$ (indicated

by **). (Student's *t*-test)

Supplementary Figure S2. Cytosolic and organellar Ca^{2+} imaging. Control vector (C)-, wild-type Bcl-2 (WT)-, and Bcl-2 mutant (mt)-overexpressing MDCK cells were loaded with (**a**) 2 μM mag-fura-2/acetoxymethyl ester (mag-fura-2/AM), (**b**) 2 μM fura-2/acetoxymethyl ester (fura-2/AM), or (**c**) 2 μM rhod-2/acetoxymethyl ester (rhod-2/AM) at 37°C for 30 min as fluorescent indicators for ER Ca^{2+} ($[Ca^{2+}]_{ER}$), cytosolic Ca^{2+} ($[Ca^{2+}]_i$), and mitochondrial Ca^{2+} ($[Ca^{2+}]_{mito}$), respectively. Representative pseudocolor images of $[Ca^{2+}]_{ER}$ and $[Ca^{2+}]_i$ were taken under a single cell fluorimeter, whereas red emission images of $[Ca^{2+}]_{mito}$ were taken under a confocal microscope. Scale bar, 20 μm.

Supplementary Figure S3. Bcl-2 inhibits basal calpain activity and prevents calpain abundance. (**a**) *t*-Boc-LM-CMAC, a fluorogenic calpain substrate, was used to indicate the calpain activity. Representative fluorescence images of *t*-Boc-LM-CMAC were taken from control vector (C)-, wild-type Bcl-2 (WT)-, and Bcl-2 mutant (mt)-overexpressing MDCK cells using confocal microscopy (scale bar, 20 μm). (**b**) Quantitative analysis of the relative fluorescence intensity of *t*-Boc-LM-CMAC compared to parental (C) cells. All values are represented as mean ± SEM (where, $n \geq 100$ cells). The data were found to be statistically significant at $p < 0.05$ (indicated by

*) and p < 0.01 (indicated by **). (n = 3; Student's t-test) (**c**) Western blotting of μ-calpain and α-spectrin was performed in Bcl-2 overexpressed MDCK cells. Pictures are representative of three independent experiments. Arrow and arrowheads indicate the full length (280 kDa) and calpain-cleaved (145 kDa and 120 kDa) α-spectrin, respectively.

Supplementary Figure S4. The Bcl-2 α5-helix modulates activity of Bcl-2 and Bax. Western immunoblotting of phosphorylated Bcl-2 at serine 70 (pSer70-Bcl-2), phosphorylated Bax at threonine 167 (pThr167-Bax), Bax, Bak, and the internal control β-actin in whole cell lysates of MDCK cells with stable overexpressed control vector (C), wild-type Bcl-2 (WT), and Bcl-2 mutant (mt).

Supplementary videos

Supplementary Video S1. Time-lapse confocal imaging of ECFP and EYFP under excitation of ECFP in Bcl-2 mutant (mt)-overexpressing MDCK cells before and after TG treatment.

Supplementary Video S2. Emission ratiometric images of EYFP and ECFP ($EYFP_{em}/ECFP_{em}$) under excitation of ECFP in Bcl-2 mutant (mt)-overexpressing MDCK before and after TG treatment by time-lapse confocal imaging.

Fig. S1

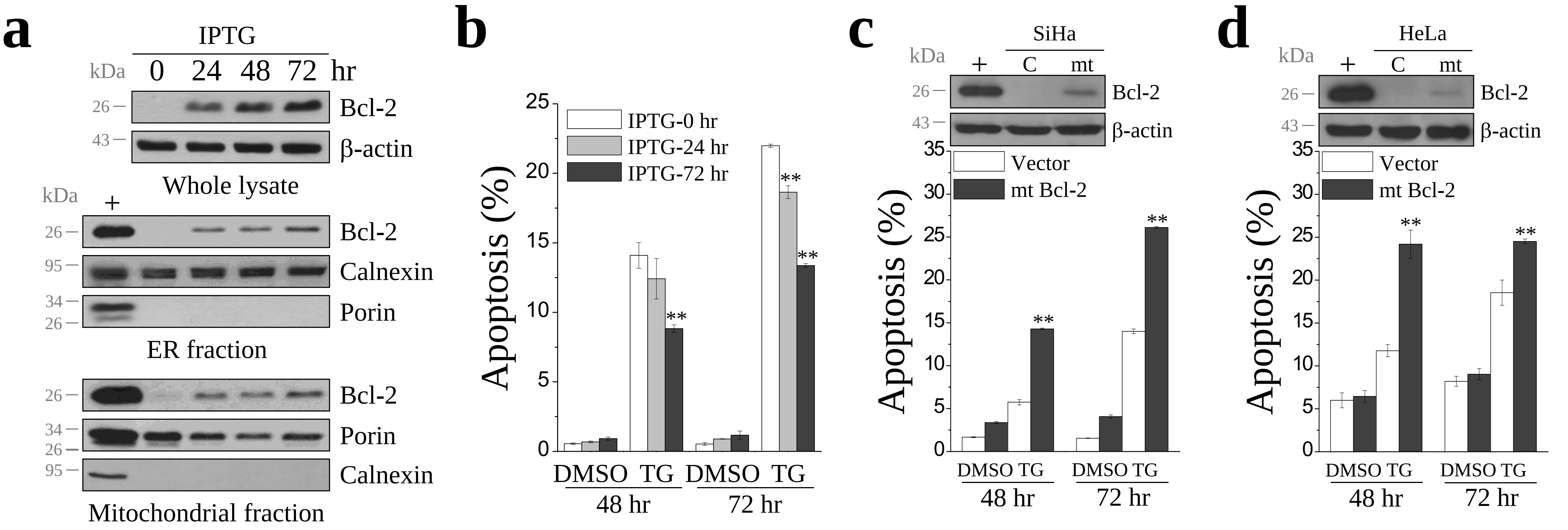
a
IPTG
kDa
0
24
48
72
hr
26
43
Bcl-2
β-actin
Whole lysate
kDa
+
26
95
34
26
Bcl-2
Calnexin
Porin
ER fraction
26
34
26
95
Bcl-2
Porin
Calnexin
Mitochondrial fraction
b
Apoptosis (%)
IPTG-0 hr
IPTG-24 hr
IPTG-72 hr
**
**
**
DMSO
TG
DMSO
TG
48 hr
72 hr
c
SiHa
kDa
+
C
mt
26
43
Bcl-2
β-actin
Vector
mt Bcl-2
Apoptosis (%)
**
**
DMSO TG
DMSO TG
48 hr
72 hr
d
HeLa
kDa
+
C
mt
26
43
Bcl-2
β-actin
Vector
mt Bcl-2
Apoptosis (%)
**
**
DMSO TG
DMSO TG
48 hr
72 hr

Fig. S2

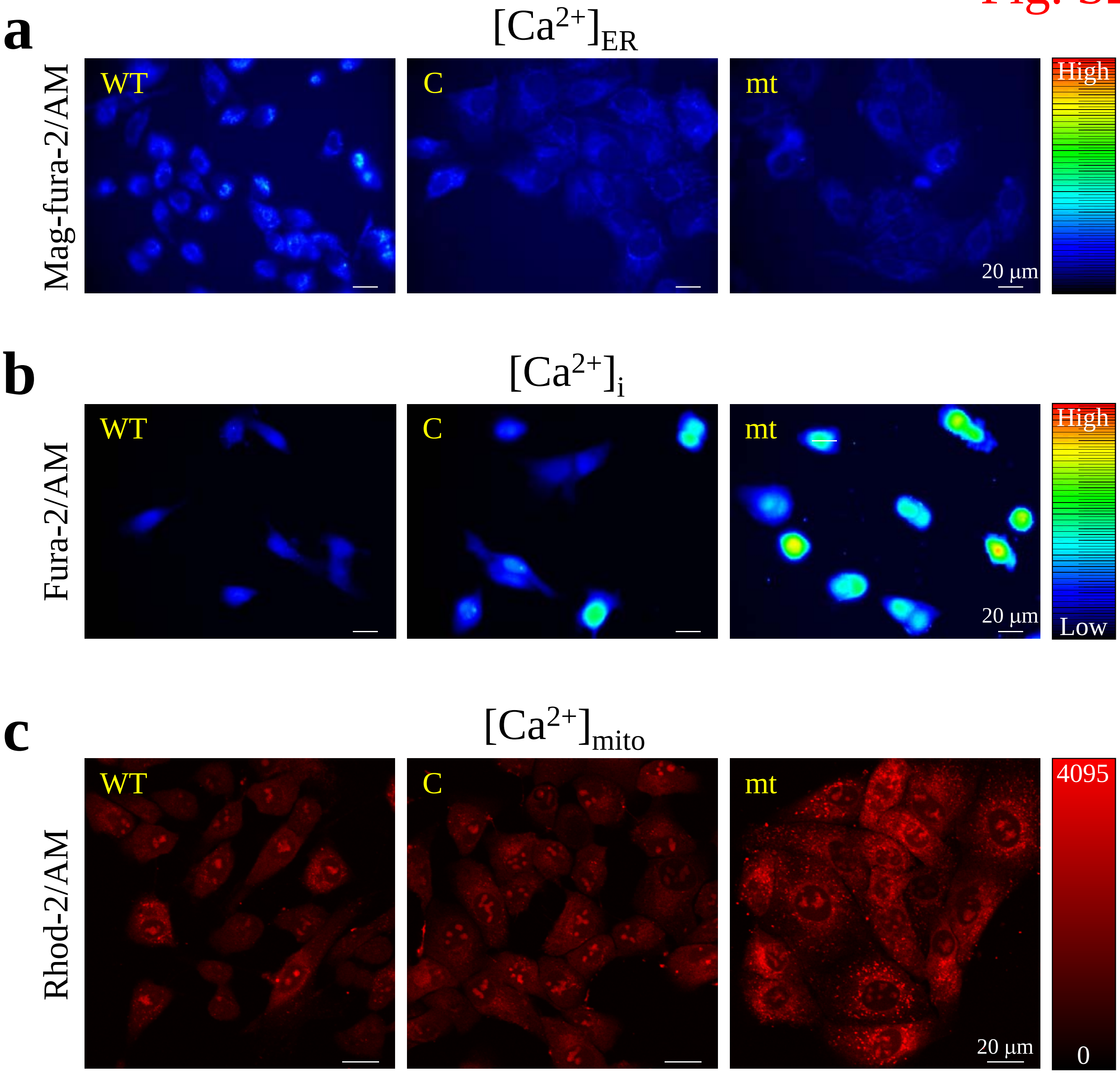

a
[Ca2+]ER
Mag-fura-2/AM
WT
C
mt
20 μm
High
b
[Ca2+]i
Fura-2/AM
WT
C
mt
20 μm
High
Low
c
[Ca2+]mito
Rhod-2/AM
WT
C
mt
20 μm
4095
0

Fig. S3

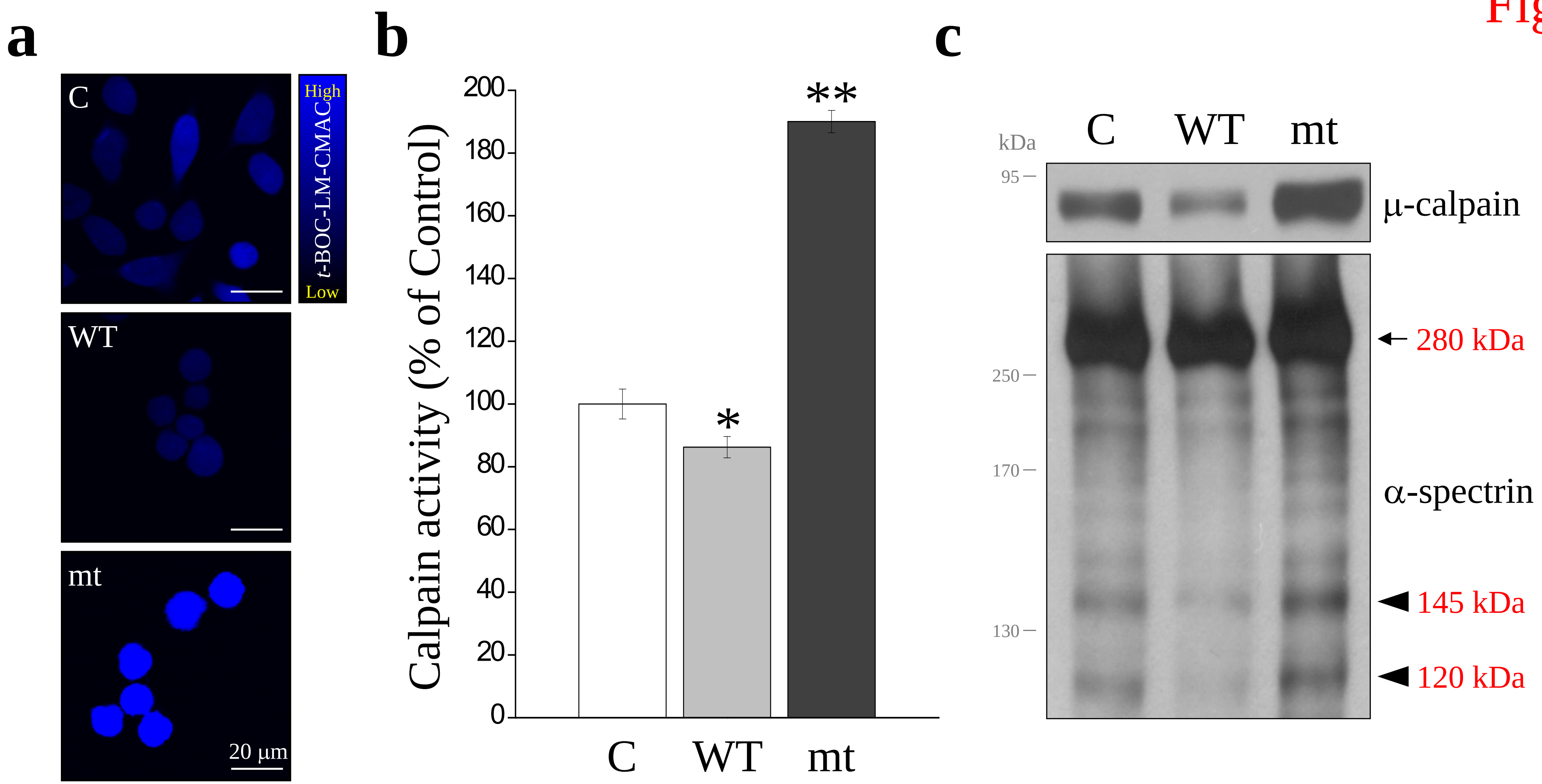
a
C
WT
mt
High
t-BOC-LM-CMAC
Low
20 µm
b
Calpain activity (% of Control)
200
180
160
140
120
100
80
60
40
20
0
*
**
C
WT
mt
c
kDa
C
WT
mt
95
250
170
130
µ-calpain
α-spectrin
280 kDa
145 kDa
120 kDa

Fig. S4

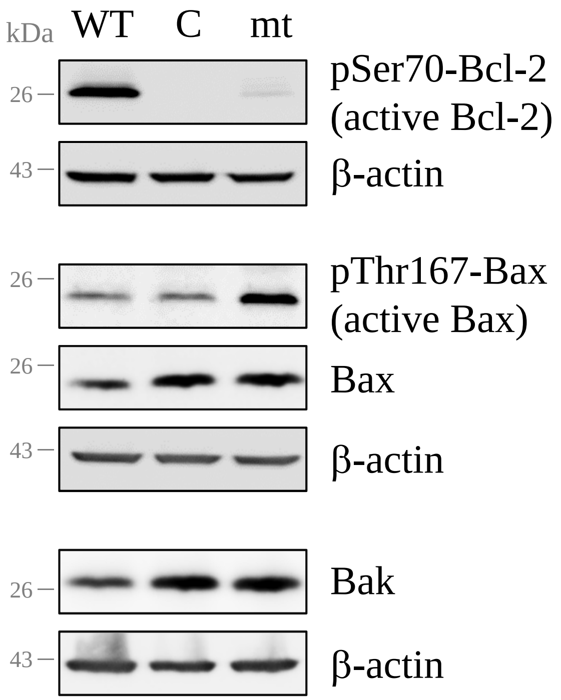
kDa
WT
C
mt
26
pSer70-Bcl-2
(active Bcl-2)
43
β-actin
26
pThr167-Bax
(active Bax)
26
Bax
43
β-actin
26
Bak
43
β-actin
